# Are People With Chronic Diseases Interested in Using Telehealth? A Cross-Sectional Postal Survey

**DOI:** 10.2196/jmir.3257

**Published:** 2014-05-08

**Authors:** Louisa Edwards, Clare Thomas, Alison Gregory, Lucy Yardley, Alicia O'Cathain, Alan A Montgomery, Chris Salisbury

**Affiliations:** ^1^Centre for Academic Primary CareSchool of Social and Community MedicineUniversity of BristolBristolUnited Kingdom; ^2^Department of PsychologyUniversity of SouthamptonSouthamptonUnited Kingdom; ^3^Medical Care Research UnitSchool of Health and Related Research (ScHARR)University of SheffieldSheffieldUnited Kingdom; ^4^Nottingham Clinical Trials UnitUniversity of NottinghamNottinghamUnited Kingdom

**Keywords:** telehealth, Internet, technology, cardiovascular diseases, depression, mental health, chronic disease, survey methodology, patient acceptance of health care

## Abstract

**Background:**

There is growing interest in telehealth—the use of technology to support the remote delivery of health care and promote self-management—as a potential alternative to face-to-face care for patients with chronic diseases. However, little is known about what precipitates interest in the use of telehealth among these patients.

**Objective:**

This survey forms part of a research program to develop and evaluate a telehealth intervention for patients with two exemplar chronic diseases: depression and raised cardiovascular disease (CVD) risk. The survey was designed to explore the key factors that influence interest in using telehealth in these patient groups.

**Methods:**

Thirty-four general practices were recruited from two different regions within England. Practice records were searched for patients with (1) depression (aged 18+ years) or (2) 10-year risk of CVD ≥20% and at least one modifiable risk factor (aged 40-74 years). Within each general practice, 54 patients in each chronic disease group were randomly selected to receive a postal questionnaire. Questions assessed five key constructs: sociodemographics, health needs, difficulties accessing health care, technology-related factors (availability, confidence using technology, perceived benefits and drawbacks of telehealth), and satisfaction with prior use of telehealth. Respondents also rated their interest in using different technologies for telehealth (phone, email and Internet, or social media). Relationships between the key constructs and interest in using the three mediums of telehealth were examined using multivariable regression models.

**Results:**

Of the 3329 patients who were sent a study questionnaire, 44.40% completed it (872/1740, 50.11% CVD risk; 606/1589, 38.14% depression). Overall, there was moderate interest in using phone-based (854/1423, 60.01%) and email/Internet-based (816/1425, 57.26%) telehealth, but very little interest in social media (243/1430, 16.99%). After adjusting for health needs, access difficulties, technology-related factors, and prior use of telehealth, interest in telehealth had largely no association with sociodemographic variables. For both patient groups and for each of the three technology mediums, the most important constructs related to interest in telehealth were having the confidence to use the associated technology, as well as perceiving greater advantages and fewer disadvantages from using telehealth. To illustrate, greater confidence using phone technologies (b=.16, 95% CI 0.002-0.33), while also perceiving more benefits (b=.31, 95% CI 0.21-0.40) and fewer drawbacks (b=-.23, 95% CI -0.28 to -0.17) to using telehealth were associated with more interest in using phone-based telehealth technologies for patients with depression.

**Conclusions:**

There is widespread interest in using phone-based and email/Internet-based telehealth among patients with chronic diseases, regardless of their health status, access difficulties, age, or many other sociodemographic factors. This interest could be increased by helping patients gain confidence using technologies and through highlighting benefits and addressing concerns about telehealth. While the same pattern exists for social media telehealth, interest in using these technologies is minimal.

## Introduction

Health care systems globally are likely to struggle to cope with the ever-increasing number of people with chronic diseases, and the United Kingdom is no exception [1,2]. For example, nearly a third of the population has a chronic disease, and this figure is projected to rise by 23% within 25 years [3]. Furthermore, health service use is high among this group of patients. Patients with chronic diseases use a large portion of general practitioner (52%) and outpatient (65%) appointments, and an estimated 69% of the primary and acute care budget is spent supporting patients with chronic diseases [3]. Therefore, exploring alternative ways of delivering care, supporting patients, and managing chronic diseases is needed, particularly in light of the financial pressures currently facing health care systems.

There is considerable international interest in telehealth as a possible alternative to face-to-face care for people with chronic diseases [2,4]. Similar to the World Health Organization [5], we define telehealth as the use of technology to support the remote delivery of health care and promote self-management. Both cost-effectiveness and clinical effectiveness should be demonstrated before telehealth becomes a mainstay in the health care system. Although some pilot studies have suggested large potential cost savings [6], a recent large randomized controlled trial of telehealth and telecare suggested that it was unlikely to be cost-effective [7]. The results from this Whole System Demonstrator trial did, however, suggest reduced mortality and emergency admission rates [8]. While this latter result is promising, some reviews conclude that the evidence for the clinical effectiveness of telehealth is, in fact, mixed [9]. It may be that some forms of telehealth can lead to improvements in patients with certain chronic diseases, such as cardiovascular disease (CVD) [10] and depression [11], since there is evidence for the effectiveness of some specific telehealth interventions in these conditions. For example, blood pressure self-monitoring, combined with self-titration of antihypertensive medication resulted in significant reductions in systolic blood pressure compared to usual care [12], while a therapist-delivered online cognitive behavioral therapy program led to greater recovery from depression than usual care [13].

Equity, as well as cost-effectiveness, is an important consideration for health care systems. Telehealth has the potential to improve care for patients with difficulty accessing traditional services, such as those who are housebound or live in rural areas [14]. These patients are also likely to be those who have the greatest health needs [15]. Additionally, since telehealth can enable a patient to monitor their own vital signs at home (eg, blood pressure), it may be more convenient and comfortable, enhance independence, and empower patients [16].

To realize the benefits of telehealth, patients must engage with and make use of it [17]. Some previous studies have suggested limited engagement with telehealth interventions in patients with chronic diseases [18], and a refusal rate of up to 75% from those invited to join telehealth trials [19]. If telehealth is to make an important contribution to the health care system for managing chronic diseases, it is imperative to identify, and then appropriately target, the factors that influence interest in telehealth, because people must be interested if they are going to make use of it. A systematic review of 52 studies on patient acceptance of computer-based health information technologies concluded that the majority of literature to date has focused on patient factors, such as sociodemographic variables [20]. For example, some previous research has suggested that interest in telehealth is highest in younger, educated, and affluent patients [21,22], but these characteristics are inversely associated with the prevalence of chronic diseases [3]. A recent review commissioned by the National Health Service (NHS) in England [22] identified five categories of barriers and facilitators to telehealth services: user characteristics, technological aspects, characteristics of services, social aspects of use, and telehealth services in use. However, both this and the aforementioned review [20] were not limited to patients with chronic diseases, nor did they aim to quantitatively assess the relative importance of factors influencing interest in telehealth. Nonetheless, in line with some of the findings from these reviews, we reasoned that both structural and evaluative factors would be key influences of interest in telehealth; namely, whether these patients have the technology readily available to use, their confidence in using technology, and their attitude towards telehealth. Moreover, if those with the greatest health needs and greatest difficulties in accessing health care are indeed interested in using telehealth, a large gap in unmet need could be filled.

We carried out a study to investigate the factors that influence interest in telehealth among patients with chronic diseases. This work was conducted as part of a larger research program exploring the potential role of an existing health service in England, NHS Direct, in providing support for chronic diseases via the telephone and Internet. For this reason, we did not name specific or existing telehealth services but asked a large number of respondents about their interest in using several types of technology that *could* be used for telehealth. The aim of the current study was to determine whether interest in telehealth among patients with chronic diseases is related to health needs, difficulties in accessing health care, or technology-related factors, including availability and attitudes to technology, while also considering the role of sociodemographic factors and taking into account prior experience using NHS Direct.

## Methods

### Design

We used a cross-sectional postal survey for this study.

### Choice of Chronic Diseases

This study focused on two exemplar chronic diseases. The first was risk of a cardiovascular event (heart attack or stroke) ≥20% over the next 10 years. This approach recognizes that hypertension, obesity, and hyperlipidemia are risk factors for CVD, rather than conditions, and it is more appropriate to consider raised CVD risk as a chronic disease [23]. The second exemplar chronic disease was depression. These two conditions were chosen to represent different types of chronic diseases, both of which are common, in which there is considerable unmet need, and where there is some evidence that particular forms of telehealth may be of clinical benefit [10-13].

### Sampling and Recruitment

General practices in two geographical areas of the United Kingdom, the south west and the north east, were invited to take part in the study. General practices were intentionally selected to represent a wide mix of socioeconomic characteristics of patients. Between August 2010 and May 2011, a query was run on practice records to identify patients with either depression (aged ≥18 years, had consulted their doctor about a mental health issue, and were prescribed an antidepressant medication within the last year) or raised risk of CVD (aged 40-74 years, QRISK2 [24] or Framingham [25] 10-year risk ≥20%, and at least one modifiable risk factor, including hypertension, obesity, or smoking). We calculated QRISK2 to assess CVD risk where possible, but since this score was not available through all general practice computer systems, we used Framingham risk scores in some practices. Patients were excluded if they were terminally ill, had cognitive impairment, or had a severe mental health condition, such as psychosis.

Fifty-four patients per practice from each of the two groups of eligible patients were selected using stratified random sampling. We sampled females and males in proportion to the number of eligible patients in each general practice. The CVD risk group was further stratified by age, such that equal proportions of young (aged 40-59 years) and older (60-74 years) participants were selected. This was because CVD risk ≥20% is more prevalent among older individuals, while access to technology is inversely associated with age [21]. Prior to invitation, general practitioners (GPs) reviewed the patient lists and excluded any patients for whom it would be inappropriate to send a questionnaire (eg, due to recent bereavement). The remaining patients were then mailed a letter by their general practice inviting them to take part in a study looking at new ways the NHS could help people to improve their health, as well as a participant information pamphlet and a questionnaire. Patients were asked to return a blank questionnaire if they did not want to take part. Those who did not respond were sent up to two postal reminders at approximately 2-week intervals. All correspondence was sent by staff at the patient’s general practice, and the researchers did not have access to patient identifiable data at any point. Ethical approval was granted by the Southmead Research Ethics Committee.

### Sample Size

Assuming an approximate 60% response rate, inviting 54 patients from each of 32 practices would provide around 960 respondents for each chronic disease group. This would provide 80% power to detect an absolute difference of ≤9.2% points in interest in using telehealth (binary outcome, equivalent odds ratio ≤1.45), with two-sided 5% alpha.

### Measures

The questionnaire included questions about the key constructs that we hypothesized would predict interest in telehealth; namely, sociodemographics, health needs, difficulties accessing health care, availability and attitudes to technology, and prior use of telehealth. In order to ensure the coherence of the questions included to assess these constructs and to reduce the questionnaire items to a smaller number of factors for data analysis, principal components analyses (PCA) with orthogonal (varimax) rotation were carried out using STATA on constructed items. Decisions regarding the number of factors to extract were based on Kaiser’s criterion (eigenvalues >1.0), by examining the scree plot and the subjective coherence of the factors. For each factor, items with an association ≥.3 were retained [26]. Next, the reliability of each factor was examined with Cronbach alpha, whereby coefficients above .70 indicate adequate reliability. Finally, mean summary scores for each reliable factor were calculated for individuals providing ratings for ≥50% of the relevant items. We treated each factor as a scale and labeled it according to the questions it comprised.

### Outcome Variable

Interest in telehealth was assessed using questions about the participant’s interest in using a range of technologies. The item reduction techniques described above resulted in three summary scores for interest in telehealth, which relate to interest in three types of technology: Phone (alpha=.82; landline or mobile phone), Email/Internet (alpha=.94; using email or doing searches on the Internet), and Social Media (alpha=.85; using chat rooms and social networking sites). These “interest” summary scores were equal to the averaged sum of responses to three question items each (range: 1-3), such that each corresponding summary score ranged from 1.0 (“Not at all interested”/“I don’t know what this is”) to 3.0 (“Very interested”), with scores of 2.0 equal to “Fairly interested”.

### Explanatory Variables

Questions about sociodemographic characteristics of the respondents included sex, ethnicity [27], age group, employment status [28], educational qualifications [29], and home ownership [30]. These questions were based on those used in previous validated surveys where possible.

Health needs were assessed using the Short-Form (SF-12v2) Health Survey, version 2 [31]. Physical Component Summary (PCS) and Mental Component Summary (MCS) scores were derived from the 12 items using proprietary scoring software (QualityMetric, Incorporated). These indexes of physical and mental health functioning are standardized with a mean of 50 and standard deviation of 10, such that lower scores indicate poorer health or greater needs.

The remainder of the questionnaire contained items constructed for the purposes of this research, although guided and informed by relevant literature, and piloted with service users in advance. These are described below, while the specific questions that comprise each scale are located in Multimedia Appendix 1.

Difficulty accessing health care was assessed using a series of questions that were based on themes identified through previous research [32,33]. Two “access difficulty” summary scores resulted: Service Delivery difficulties (7 items, alpha=.87) included questions about the convenience of accessing health care, as well as the nature or quality of the care itself (eg, getting the right amount of care), and Physical Access difficulties (4 items, alpha=.78) included questions about trouble getting to appointments due to physical, psychological, and transport problems, including cost. These summary scores ranged between 1.0 (“No difficulty”) and 3.0 (“Lots of difficulty”).

Technology-related factors included questions about availability of technologies and attitudes towards telehealth. Technology availability was assessed by asking respondents which of a range of technologies were easily available for them to use. Phone Availability (2 items: landline, mobile) and Email/Internet Availability (2 items: have email address, Internet access) scores were formed by summing tallies (0=Absent, 1=Present) for these technologies.

Questions about attitudes towards telehealth were based on the theory of planned behavior [34]. This theory suggests that perceived behavioral control—a concept capturing the extent to which one believes one is able to perform a behavior—directly influences one’s intention to carry out a behavior and may predict behavior itself. Beliefs about one’s capability, which should be reflected in confidence levels, affect perceived behavioral control. Therefore, questions about confidence using different types of technology were devised. After item reduction, there were three clusters representing confidence in using Phone-based technologies (3 items, alpha=.74), Email/Internet-based technologies (3 items, alpha=.96), and Social Media-based technologies (3 items, alpha=.88). Again, larger scores indicate greater technology confidence [range: 1.0 (“Not at all confident”/“I have never tried this”/“I don’t know what this is”) to 3.0 (“Extremely confident”)].

The theory of planned behavior also states that positive or negative attitudes towards a behavior predict one’s intention to perform that behavior and are influenced by beliefs about the advantages and disadvantages of that behavior [34]. Hence, items about the potential advantages and disadvantages of telehealth were generated based on previous qualitative research [35]. Summary scores for telehealth Advantages (7 items, alpha=.87) and Disadvantages (7 items, alpha=.90) were similarly formed (range: 1.0 (“Strongly disagree”) to 5.0 (“Strongly agree”); higher scores reflect greater perceived advantages and disadvantages.

Finally, satisfaction with prior use of telehealth that was delivered by NHS Direct was evaluated by a single item. Respondents rated how satisfied they were with previous use of NHS Direct services on a scale from 1 (“Not at all”) to 5 (“Extremely”). NHS Direct is a service freely available throughout England that provides health assessments, information, and advice. It currently provides telehealth via telephone and its interactive website, but NHS Direct and other similar services could act as a provider of a wider range of telehealth services.

### Statistical Analysis

The primary analysis investigated the extent to which interest in the use of telehealth was related to five key constructs: sociodemographic factors, health needs (including physical and mental health), access difficulties (including service delivery and physical access), technology-related factors (availability of technology and attitudes towards telehealth), and satisfaction with prior use of telehealth. We first used appropriate descriptive statistics (mean and SD, or n and %) to summarize the sociodemographic characteristics of respondents, and their needs, access difficulties, technology factors, and interest in using telehealth. This included an exploration of how needs, access, and technology factors varied by age and chronic disease group. We then used multivariable regression models to examine associations between these variables and interest in telehealth, adjusting for the other variables in the model, and taking into account the stratified survey design.

## Results

### Response Rate

Thirty-four general practices took part in the survey. GPs excluded 5.23% of the CVD risk group (96/1836) and 11.23% of patients with depression (201/1790) prior to mailing questionnaires. Of the 3329 patients who were sent a study questionnaire, 1478 (44.40%) returned it. The response rate was higher for patients with CVD risk (872/1740, 50.11%) than for depression (606/1589, 38.14%). Separate logistic regression analyses for the two patient groups revealed that response rates for both CVD risk and depression were higher in older people, whereas the likelihood of responding did not differ by respondent sex or location (Table 1).

**Table 1:** Demographic differences between responders and non-responders by patient group.

Characteristics		Patient group: CVD risk (n=1635)			Patient group: depression (n=1497)		
			Responded (n=828)	No response (n=807)		Responded (n=583)	No response (n=914)
		OR (95% CI)	n (%)	n (%)	OR (95% CI)	n (%)	n (%)
**Age (years)**							
	18-29	−	−	−	Referent	64 (11.0)	218 (23.9)
	30-44	Referent^a^	18 (2.2)	39 (4.8)	2.0 (1.4-2.8)	166 (28.5)	311 (34.0)
	45-59	1.6 (0.6-3.9)	290 (35.0)	391 (48.5)	3.5 (2.5-5.0)	197 (33.8)	232 (25.4)
	60-74	3.0 (1.2-7.2)	514 (62.1)	377 (46.7)	4.4 (3.0-6.4)	112 (19.2)	90 (9.8)
	75+	−	6 (0.7)	0 (0.0)	2.8 (1.6-5.0)	44 (7.5)	63 (6.9)
**Sex** ^b^		1.3 (0.9-1.7)			1.4 (0.9-1.9)		
	Male		620 (74.9)	621 (77.0)		148 (25.4)	295 (32.3)
	Female		208 (25.1)	186 (23.0)		435 (74.6)	619 (67.7)
**Location** ^c^		0.8 (0.6-1.0)			1.0 (0.8-1.4)		
	Bristol		438 (52.9)	386 (47.8)		282 (48.4)	472 (51.6)
	Sheffield		390 (47.1)	421 (52.2)		301 (51.6)	442 (48.4)

^a^Referent age group is 40-44 years.

^b^Sex (0=Male, 1=Female).

^c^Location (0=Bristol, 1=Sheffield).

### Sample Characteristics

Patients with CVD risk (mean 61.9 years, SD 7.8) were older than those with depression (mean 49.1 years, SD 15.9), reflecting the inclusion criteria. Three-quarters of the CVD risk group were male (654/872, 75.0%), while three-quarters of the depression group were female (452/606, 74.6%). Both patient groups were predominantly Caucasian (CVD 825/851, 96.9%; depression 575/594, 96.8%), most were not currently employed (unemployed, studying, retired, etc) (CVD 498/861, 57.8%; depression 317/597, 53.1%), only a minority had higher education (CVD 212/872, 24.3%; depression 222/606, 36.6%), while the majority were home owners (CVD 647/859, 75.3%; depression 410/595, 68.9%).

### Overview of Health Needs, Access Difficulties, and Technology-Related Factors

As expected, patients with CVD risk reported poorer physical than mental health, whereas the reverse was true of patients with depression (Table 2). While the reported physical health of patients with CVD risk was half a standard deviation below that of the national average (UK mean 50.9, SD 9.4), the reported mental health of patients with depression was more than 1.5 standard deviations below this average (UK mean 50.9, SD 9.4) [36].

Few patients reported access difficulties, with all summary scores approximating the “No difficulty” response category (Table 2). Despite these low mean summary scores, an important minority of participants indicated some difficulty in accessing health care, and both patient groups were more likely to report having service delivery than physical access difficulties. For example, 27.86% (399/1432) of patients reported difficulties getting care when they need it most (service delivery), while 14.23% (206/1448) reported difficulties traveling to appointments due to physical health (physical access).

Technology availability was high across both patient groups (Table 2). Phone technologies were more prevalent than computer-based technologies and markedly so for the patients with CVD risk. In fact, nearly all patients had access to phone technologies. Across patient groups, age was associated only with availability of the computer-based technologies: 90.0% (115/128) of the two youngest age groups (18-44 years), 78.1% (400/512) of those aged 45-59 years, and 60.5% (393/650) of those aged 60-74 years reported that they have these technologies readily available to use. It is among only the oldest, and proportionally smallest, age group (n=49) that less than half the respondents (13, 26.5%) report easy access to computer technologies.

**Table 2:** Health needs, access difficulties, technology-related factors, and satisfaction with prior telehealth use by patient group.

Characteristics		Patient group: CVD risk	Patient group: depression
**Health needs, mean (SD), n**			
	PCS	45.3 (11.8), 777	47.3 (13.8), 547
	MCS	49.8 (10.5), 777	37.7 (12.9), 547
**Access difficulties, mean (SD), n** ^a^			
	Service delivery difficulties	1.3 (0.4), 848	1.5 (0.5), 595
	Physical access difficulties	1.1 (0.3), 854	1.2 (0.4), 594
**Technology-related factors**			
	Phone availability, % (n)^b^	98.4 (855/869)	99.3 (595/599)
	Email/Internet availability, % (n)^b^	67.2 (584/869)	80.3 (481/599)
	Phone confidence, mean (SD), n^c^	2.5 (0.6), 861	2.5 (0.6), 596
	Email/Internet confidence, mean (SD), n^c^	2.0 (0.9), 851	2.3 (0.8), 595
	Social media confidence, mean (SD), n^c^	1.3 (0.6), 847	1.6 (0.8), 594
	Telehealth advantages, mean (SD), n^d^	3.6 (0.8), 853	3.7 (0.7), 588
	Telehealth disadvantages, mean (SD), n^d^	3.5 (0.9), 860	3.3 (0.9), 593
**Satisfaction with prior telehealth use, mean (SD), n** ^e^			
	NHS Direct satisfaction	3.4 (1.2), 247	3.4 (1.2), 336

^a^Range: 1.0-3.0, where higher scores indicate greater access difficulties. Service delivery difficulties included questions about the convenience of accessing health care, as well as the nature or quality of the care itself, eg, getting the right amount of care. Physical access difficulties included questions about trouble getting to appointments due to physical, psychological, and transport problems, including cost, as detailed in the Methods section.

^b^Technology availability includes having one or more forms of relevant technology.

^c^Range: 1.0-3.0, where higher scores indicate greater technology confidence.

^d^Range: 1.0-5.0, where higher scores indicate greater perceived advantages and disadvantages of telehealth.

^e^Range: 1.0-5.0, where higher scores indicate greater satisfaction with past NHS Direct use.

**Figure 1:**
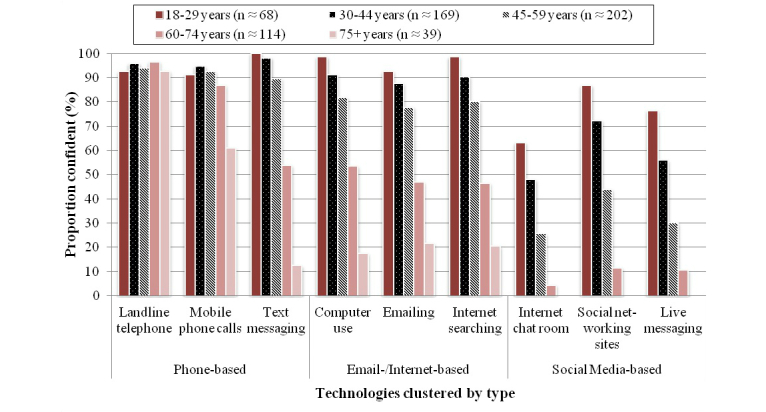
Proportion of depression respondents within each age group reporting confidence using individual technologies.

Technology confidence ratings were similar between patient groups, but they varied somewhat across the technology types (Table 2) and age groups (Figure 1). In general, patients reported greatest confidence using phone technologies, with mean summary scores approaching the “Extremely confident” response category, and least confidence using the social media technologies, with mean summary scores close to “Not at all confident”. Respondents were “Quite confident” with the email-based and Internet-based technology category.

Figure 1 shows the proportion of depression respondents reporting they were “Extremely confident” or “Quite confident” using the various technologies across the different age groups. The pattern of findings was similar among the CVD risk group (data not shown). Least associated with age were the phone technologies, which received high confidence ratings by all age groups. The one exception was low confidence in text messaging by the oldest age group. Although confidence using email/Internet and social media technologies consistently decreased with age, more than half of the respondents in all age groups (except the over-75s) reported confidence using email/Internet technologies. Conversely, confidence using social media technologies was strongly related to age, with only the younger age groups expressing confidence.

Summary scores indicate similar levels of perceived advantages and disadvantages of using telehealth across patient groups (Table 2). The most highly endorsed advantages were convenience and ability of telehealth to be delivered when and where one desires (Table 3). Dislike of non–face-to-face care and concerns over security issues emerged as the top disadvantages of telehealth (Table 3).

Of those respondents that had ever used NHS Direct (Table 2), the majority were satisfied with that experience: 26.9% (157/583) were “Moderately”, 33.1% (193/583) were “Quite a bit”, and 18.9% (110/583) were “Extremely” satisfied.

**Table 3:** Proportion of respondents agreeing^a^with each of the potential advantages and disadvantages of using telehealth by patient group.

		Patient group: CVD risk, % (n)	Patient group: depression, % (n)
**Advantages**			
	I would like being able to choose to get support at times that are best for me.	81.7 (696/852)	87.4 (514/588)
	I would find it reassuring to be able to get support when I feel that I need it most.	81.0 (689/851)	85.7 (504/588)
	I would like being able to get support in my own home.	67.7 (573/846)	71.2 (418/587)
	Getting support with my health by phone or computer would be valuable to me.	54.9 (466/849)	60.8 (360/592)
	I could save money by not having to travel to appointments.	50.7 (426/840)	51.4 (299/582)
	Getting support in this way would help me to feel more independent.	48.8 (413/847)	54.2 (318/587)
	It would make me feel special to be getting ‘extra’ support when I feel that I need it most.	41.9 (354/844)	42.5 (247/581)
**Disadvantages**			
	I would dislike being unable to see the person face-to-face.	66.6 (571/858)	60.2 (357/593)
	I would be concerned about the security of the information that I give.	63.3 (544/860)	60.3 (357/592)
	I would not want to discuss sensitive issues over the phone or using a computer	61.9 (532/859)	54.9 (325/592)
	I would dislike speaking to someone other than a doctor about my health.	53.7 (462/860)	45.8 (271/592)
	I would worry about relying too much on the technology.	52.3 (447/854)	42.2 (247/586)
	I would worry about the possibility of the equipment not working.	45.4 (387/852)	37.8 (222/588)
	Getting support in this way would make me feel anxious about my health.	33.2 (284/855)	26.0 (153/588)

^a^A response of either “Strongly agree” or “Agree” on a 5-point scale was considered agreement.

### Overview of Interest in Using Telehealth

Regardless of patient group, there was moderate interest in phone (CVD risk mean 1.7, SD 0.6; depression mean 1.9, SD 0.7) and email/Internet technologies (CVD risk mean 1.7, SD 0.7; depression mean 1.9, SD 0.7). These mean summary scores approximate the “Fairly interested” response category. In contrast, there was very little interest in the social media technologies (CVD risk mean 1.2, SD 0.4; depression mean 1.3, SD 0.5).

**Figure 2:**
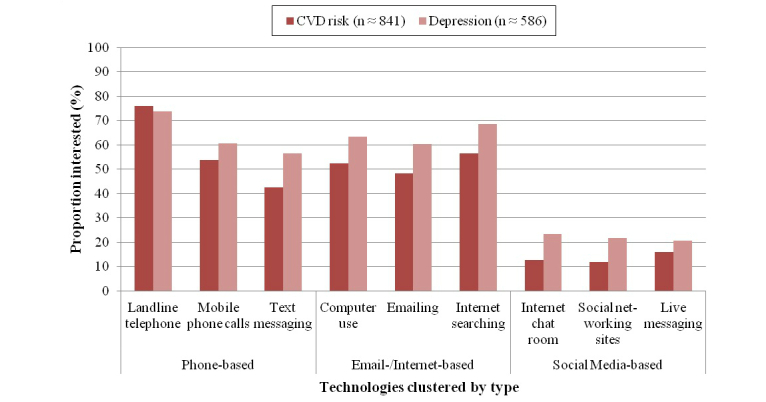
Proportion of respondents interested in individual telehealth technologies by patient group.

Figure 2 shows which individual technologies respondents were more or less interested in using, with ratings of interest dichotomized into some versus no interest for ease of interpretation. This shows that patients with depression were more interested than those with CVD risk in nearly every form of technology for telehealth. There was a clear preference for the landline telephone (1072/1428, 75.07% overall), followed by finding information on the Internet (876/1427, 61.39% overall). Again, there was hardly any interest in the social media technologies. Averaging across the technology types and both patient groups (CVD risk and depression), there was moderate interest in using phone-based (854/1423, 60.01%) and email/Internet-based (816/1425, 57.26%) telehealth, but very little interest in social media (243/1430, 16.99%).

### What Factors are Associated With Interest in Telehealth?

To address the main research question, sociodemographics, health needs, access difficulties, technology-related factors, and satisfaction with prior telehealth use were simultaneously regressed on interest for each of the three telehealth mediums—phone-based, email/Internet-based, and social media–based telehealth—separately for each patient group (CVD risk and depression). From these multivariable linear regression analyses, three variables were reliably related to interest in telehealth: greater technology confidence, and perceiving both greater advantages and fewer disadvantages of telehealth were associated with more interest in using telehealth. Moreover, these factors were consistently related to interest in each of the three telehealth mediums for both patient groups (Tables 4-6). Importantly, however, the technology confidence finding is modality-specific. This means that greater phone confidence is associated with greater phone-based telehealth interest, greater email/Internet confidence is associated with greater email/Internet-based telehealth interest, while greater confidence using social media technologies is associated with greater interest in social media–based telehealth. Therefore, while confidence using a particular type of technology discriminated between interest in different modes of telehealth, perceiving greater benefits of and fewer drawbacks to using telehealth was uniformly related to greater interest.

Three other consistent findings emerged. First, for patients with depression but not those with CVD risk, greater difficulties with getting convenient, high-quality care (service delivery aspects of access) were related to more interest in phone-based and email/Internet-based telehealth technologies. Second, as anticipated, greater satisfaction with previous use of NHS Direct was associated with heightened interest in future use of phone-based telehealth, and this was consistent across both patient groups. Third, there was more interest in email/Internet-based and social media–based telehealth among those with CVD risk who were not home owners. Apart from these findings, the remaining variables in the model were unimportant to telehealth interest. After adjusting for the other variables in the model, health needs, access difficulties, technology availability, and even sociodemographic factors did not reliably and consistently have an independent effect on interest in telehealth, either across patient groups or across more than one telehealth type within a patient group.

**Table 4:** Multivariable analysis of factors associated with interest in phone-based telehealth^a^.

Characteristics			Patient group: CVD risk (n=676)		Patient group: depression (n=489)	
			b (95% CI)	*P*	b (95% CI)	*P*
**Sociodemographic factors**						
	**Age group (years)**					
		18-29	−	−	Referent	
		30-44	Referent (40-44 years)		-.054 (-0.187 to 0.079)	
		45-59	-.101 (-0.379 to 0.178)		-.016 (-0.201 to 0.168)	
		60-74	-.122 (-0.421 to 0.176)		-.028 (-0.178 to 0.122)	
		75+	.301 (-0.484 to 1.086)	.35^b^	.087 (-0.303 to 0.476)	.79^b^
	Sex^c^		-.018 (-0.122 to 0.086)	.72	-.104 (-0.226 to 0.018)	.09
	Ethnicity^d^		-.061 (-0.312 to 0.191)	.63	.161 (-0.076 to 0.398)	.18
	Employed^e^		.108 (0.015 to 0.201)	.02	.049 (-0.056 to 0.155)	.35
	Higher education^f^		-.009 (-0.092 to 0.074)	.83	-.157 (-0.272 to -0.042)	.01
	Home owner^g^		-.088 (-0.206 to 0.030)	.14	-.173 (-0.281 to -0.065)	.003
	Location^h^		-.017 (-0.123 to 0.090)	.75	-.072 (-0.170 to 0.026)	.14
**Health needs**						
	PCS		.001 (-0.003 to 0.005)	.67	.005 (0.001 to 0.009)	.02
	MCS		-.002 (-0.006 to 0.001)	.21	.004 (0.001 to 0.008)	.02
**Access difficulties** ^i^						
	Service delivery		-.093 (-0.232 to 0.046)	.19	.205 (0.069 to 0.340)	.004
	Physical access		.185 (-0.010 to 0.379)	.06	-.066 (-0.280 to 0.148)	.53
**Technology-related factors**						
	Phone availability^j^		.107 (-0.039 to 0.254)	.15	.203 (0.025 to 0.382)	.03
	Email/Internet availability^j^		-.012 (-0.118 to 0.095)	.82	-.089 (-0.178 to 0.0003)	.05
	Phone confidence^i^		.254 (0.151 to 0.358)	<.001	.164 (0.002 to 0.326)	.048
	Email/Internet confidence^i^		-.075 (-0.197 to 0.046)	.22	-.011 (-0.111 to 0.088)	.82
	Social media confidence^i^		.065 (-0.018 to 0.147)	.12	-.025 (-0.110 to 0.060)	.56
	Telehealth advantages^i^		.296 (0.240 to 0.352)	<.001	.308 (0.213 to 0.404)	<.001
	Telehealth disadvantages^i^		-.201 (-0.261 to -0.140)	<.001	-.226 (-0.282 to -0.170)	<.001
**Past telehealth satisfaction** ^i^						
	NHS Direct		.088 (0.025 to 0.151)	.01	.046 (0.001 to 0.090)	.045

^a^The associations have been adjusted by all other variables in this fully adjusted model, and the stratified survey design has been taken into account in the analysis. Interest in phone-based telehealth scores range from 1.0-3.0, with higher scores indicating more interest.

^b^Indicates *P* value from Wald test.

^c^Sex (0=Male, 1=Female).

^d^Ethnicity (0=Non-Caucasian, 1=Caucasian).

^e^Employed (0=Not employed, 1=Employed).

^f^Higher Education (0=No higher education, 1=Some higher education).

^g^Home Owner (0=Non-home owner, 1=Home owner).

^h^Location (0=Bristol, 1=Sheffield).

^i^Higher scores indicate greater access difficulties, technology confidence, advantages, disadvantages, and satisfaction.

^j^Technology Availability (0=Not available, 1=One form available, 2=Both available).

**Table 5:** Multivariable analysis of factors associated with interest in email/Internet-based telehealth^a^.

Characteristics			Patient group: CVD risk (n=681)		Patient group: depression (n=488)	
			b (95% CI)	*P*	b (95% CI)	*P*
**Sociodemographic factors**						
	**Age group (years)**					
		18-29	−	−	Referent	
		30-44	Referent (40-44 years)		-.041 (-0.278 to 0.197)	
		45-59	-.184 (-0.373 to 0.005)		.006 (-0.198 to 0.209)	
		60-74	-.132 (-0.346 to 0.082)		.045 (-0.160 to 0.250)	
		75+	.371 (-0.120 to 0.863)	.01^b^	.032 (-0.299 to 0.364)	.89^b^
	Sex^c^		-.035 (-0.122 to 0.052)	.42	.008 (-0.114 to 0.130)	.90
	Ethnicity^d^		.071 (-0.133 to 0.276)	.48	-.042 (-0.221 to 0.138)	.64
	Employed^e^		.086 (-0.010 to 0.182)	.08	-.048 (-0.146 to 0.049)	.32
	Higher education^f^		.070 (-0.068 to 0.207)	.31	-.037 (-0.155 to 0.080)	.52
	Home owner^g^		-.148 (-0.266 to -0.031)	.02	-.059 (-0.158 to 0.040)	.24
	Location^h^		-.002 (-0.096 to 0.092)	.97	-.090 (-0.164 to -0.016)	.02
**Health needs**						
	PCS		-.002 (-0.007 to 0.003)	.44	.003 (-0.001 to 0.008)	.16
	MCS		-.006 (-0.013 to 0.001)	.08	-.005 (-0.010 to 0.0004)	.07
**Access difficulties** ^i^						
	Service delivery		-.013 (-0.124 to 0.098)	.81	.087 (0.005 to 0.168)	.04
	Physical access		.017 (-0.249 to 0.284)	.90	-.127 (-0.240 to -0.013)	.03
**Technology-related factors**						
	Phone availability^j^		-.099 (-0.211 to 0.012)	.08	.093 (-0.095 to 0.282)	.32
	Email/Internet availability^j^		.158 (0.091 to 0.225)	<.001	.101 (-0.013 to 0.215)	.08
	Phone confidence^i^		-.021 (-0.121 to 0.079)	.68	-.199 (-0.367 to -0.032)	.02
	Email/Internet confidence^i^		.304 (0.219 to 0.389)	<.001	.403 (0.295 to 0.512)	<.001
	Social media confidence^i^		-.002 (-0.157 to 0.154)	.98	.003 (-0.075 to 0.080)	.95
	Telehealth advantages^i^		.226 (0.165 to 0.286)	<.001	.237 (0.150 to 0.324)	<.001
	Telehealth disadvantages^i^		-.244 (-0.310 to -0.179)	<.001	-.211 (-0.287 to -0.134)	<.001
**Past telehealth satisfaction** ^i^						
	NHS Direct		.029 (-0.036 to 0.094)	.38	.040 (-0.034 to 0.115)	.28

^a^The associations have been adjusted by all other variables in this fully adjusted model, and the stratified survey design has been taken into account in the analysis. Interest in email/Internet-based telehealth scores range from 1.0-3.0, with higher scores indicating more interest.

^b^Indicates *P* value from Wald test.

^c^Sex (0=Male, 1=Female).

^d^Ethnicity (0=Non-Caucasian, 1=Caucasian).

^e^Employed (0=Not employed, 1=Employed).

^f^Higher Education (0=No higher education, 1=Some higher education).

^g^Home Owner (0=Non-home owner, 1=Home owner).

^h^Location (0=Bristol, 1=Sheffield).

^i^Higher scores indicate greater access difficulties, technology confidence, advantages, disadvantages, and satisfaction.

^j^Technology Availability (0=Not available, 1=One form available, 2=Both available).

**Table 6:** Multivariable analysis of factors associated with interest in social media–based telehealth^a^.

Characteristics			Patient group: CVD risk (n=680)		Patient group: depression (n=489)	
			b (95% CI)	*P*	b (95% CI)	*P*
**Sociodemographic factors**						
	**Age group (years)**					
		18-29	−	−	Referent	
		30-44	Referent (40-44 years)		.146 (-0.087 to 0.379)	
		45-59	-.092 (-0.301 to 0.117)		.187 (-0.036 to 0.411)	
		60-74	-.030 (-0.246 to 0.186)		.174 (-0.033 to 0.382)	
		75+	.047 (-0.286 to 0.380)	.25^b^	.061 (-0.163 to 0.285)	.31^b^
	Sex^c^		-.025 (-0.080 to 0.030)	.36	.029 (-0.061 to 0.118)	.52
	Ethnicity^d^		-.046 (-0.255 to 0.164)	.66	.036 (-0.208 to 0.279)	.77
	Employed^e^		.040 (-0.031 to 0.110)	.26	-.007 (-0.114 to 0.100)	.90
	Higher eEducation^f^		.018 (-0.057 to 0.093)	.63	-.033 (-0.147 to 0.080)	.55
	Home owner^g^		-.118 (-0.198 to -0.038)	.01	-.097 (-0.232 to 0.038)	.15
	Location^h^		-.015 (-0.061 to 0.032)	.53	-.005 (-0.082 to 0.073)	.91
**Health needs**						
	PCS		-.001 (-0.004 to 0.001)	.28	-.001 (-0.006 to 0.004)	.67
	MCS		-.002 (-0.007 to 0.003)	.44	-.001 (-0.007 to 0.004)	.63
**Access difficulties** ^i^						
	Service delivery		-.079 (-0.161 to 0.002)	.06	.029 (-0.098 to 0.156)	.64
	Physical access		.029 (-0.118 to 0.176)	.70	-.070 (-0.200 to 0.060)	.28
**Technology-related factors**						
	Phone availability^j^		-.109 (-0.256 to 0.039)	.14	.077 (-0.096 to 0.250)	.37
	Email/Internet availability^j^		.016 (-0.029 to 0.062)	.48	.013 (-0.073 to 0.099)	.76
	Phone confidence^i^		.012 (-0.071 to 0.095)	.78	-.068 (-0.218 to 0.082)	.36
	Email/Internet confidence^i^		.001 (-0.056 to 0.057)	.98	-.038 (-0.118 to 0.041)	.33
	Social media confidence^i^		.243 (0.132 to 0.355)	<.001	.361 (0.282 to 0.441)	<.001
	Telehealth advantages^i^		.096 (0.045 to 0.146)	.001	.176 (0.106 to 0.245)	<.001
	Telehealth disadvantages^i^		-.072 (-0.128 to -0.016)	.01	-.123 (-0.191 to -0.054)	.001
**Past telehealth satisfaction** ^i^						
	NHS Direct		.033 (-0.006 to 0.072)	.10	-.006 (-0.051 to 0.040)	.80

^a^The associations have been adjusted by all other variables in this fully adjusted model, and the stratified survey design has been taken into account in the analysis. Interest in social media–based telehealth scores range from 1.0-3.0, with higher scores indicating more interest.

^b^Indicates *P* value from Wald test.

^c^Sex (0=Male, 1=Female).

^d^Ethnicity (0=Non-Caucasian, 1=Caucasian).

^e^Employed (0=Not employed, 1=Employed).

^f^Higher Education (0=No higher education, 1=Some higher education).

^g^Home Owner (0=Non-home owner, 1=Home owner).

^h^Location (0=Bristol, 1=Sheffield).

^i^Higher scores indicate greater access difficulties, technology confidence, advantages, disadvantages, and satisfaction.

^j^Technology Availability (0=Not available, 1=One form available, 2=Both available).

## Discussion

### Principal Findings

Patients with two very different chronic diseases are interested in using phone-based and email or Internet-based telehealth, but not in telehealth via social media websites. Interest in all three forms of telehealth appears to stem from the perceived advantages and disadvantages of telehealth, as well as confidence using the relevant technology. This is significant because beliefs and levels of confidence are far more malleable than most of the other constructs included in this study, such as technology availability or socioeconomic status. It is also noteworthy that these other constructs were not consistently and independently associated with interest in telehealth. First, interest in telehealth was not reliably related to health needs. This suggests that willingness to use telehealth spans across those with good and poor health. Furthermore, it is not only those who have difficulty accessing traditional health care who are motivated to use telehealth—those with and without access difficulties were interested. Sociodemographic factors were generally not, in themselves, systematically related to telehealth interest. Therefore, older people are just as interested as their younger counterparts, after adjusting for other factors, such as confidence in using the technology. While availability of technology was quite high, this factor did not consistently relate to interest in telehealth either. The ramifications of these findings are important for policy makers, researchers, health professionals, and patients alike.

### Strengths and Limitations

The main strength of this research is that it is a broad exploration of patient interest in several general forms of telehealth. The findings, therefore, are not limited to a specific intervention but highlight some of the key elements we must pay particular attention to when designing and implementing future telehealth initiatives. This is also the only study, to our knowledge, that has gathered ratings about interest in using a variety of forms of telehealth from patients that are the most likely recipients of this type of care in the future—those with chronic diseases. If telehealth is going to have an important role in effectively supporting patients with chronic diseases, then knowing what interests patients in taking up different forms of telehealth is an important first step.

The primary limitation of this study is the response rate of 44.4%, although it compares favorably with other community surveys [37]. It is possible that those who did not respond had different characteristics from those who chose to respond, which could have implications for the findings. With respect to telehealth, non-response bias by age is important given the widely held perception that older people do not like or use technology much. In this survey, responders were actually older than non-responders, and yet a fair amount of interest in telehealth was reported. There was also considerable variation in patient health and sociodemographics, as well as the other key variables of interest. These findings, nevertheless, may not be generalizable to other chronic disease patient groups. A second limitation is that the telehealth interest ratings are based on questions about hypothetical and general technologies, rather than existing or specifically named telehealth services. While this approach was directly in line with the purpose of the research—to inquire about future interest in services that *could* be delivered by existing health care providers—it is difficult to know what types of applications respondents were thinking about when they gauged their interest in telehealth delivered via social networking websites, for instance. Moreover, it is likely that there is relationship between how frequently technology is used and technology confidence [38,39], and this relationship should be controlled for in future research. Finally, the large number of variables analyzed raises the possibility of type 1 error due to multiple comparisons. Therefore, we have conservatively drawn attention only to findings that were consistent across several analyses.

### Conclusions

We examined whether those with greater health needs or greater difficulties accessing traditional health care were more interested in using telehealth but found only weak evidence for this in patients with depression. Our results revealed an association between greater service delivery access difficulties (getting the right amount of care, from the right health professional, at the right time) and heightened interest in both phone-based and email/Internet-based telehealth among patients with depression. This finding aligns well with one aim of telehealth treatments for depression; namely, overcoming barriers to care. Given the results of systematic reviews that showed that telephone-administered psychotherapy [40] attrition rates are far lower than those of face-to-face care [41], this level of engagement may suggest that telehealth meets this aim to some extent. It is important to note, however, that respondents in our survey reported very few difficulties with health care access and did not report especially great health needs, except for the mental health needs of those with depression. Overall, this restriction in range may have made it difficult to detect an effect of need or a more widespread effect of access difficulties on telehealth interest.

Sociodemographic factors were found to be relatively unimportant after adjusting for attitudes towards telehealth and availability of technology, which suggests that telehealth appeals to a broader demographic than young, educated, and affluent patients. While this runs contrary to some previous literature [21,22], it might be explained by the fact that more proximal variables, like technology confidence and beliefs about telehealth, had not been included in previous research. Indeed, when similar behavioral or motivational factors are assessed, other research is consistent with our findings in demonstrating the integral role [42], or even superiority [39], of these constructs over demographic variables, albeit in terms of using the Internet alone. Furthermore, a systematic review concluded that focusing on patient factors alone, as the majority of research in this area has done, is probably not comprehensive enough to understand patient interest in using telehealth [20]. We agree with this review that future research must cut across a broader spectrum of factors, especially those at the level of human-technology interaction, the health care system, and other social or normative influences.

Technology confidence is an example of a human-technology interaction variable, and a key finding of this and other research [20] is that confidence is consistently associated with interest in using telehealth. While we asked respondents about technology confidence, it is interesting to note that other studies have used negative framing and asked about technology anxiety [38,43] or difficulty using the Internet [39]. Nonetheless, these studies also showed the equivalent association between lower technology anxiety and heightened interest in using telehealth.

There are several interesting implications of the finding that telehealth interest is most strongly associated with technology confidence and perceived advantages and disadvantages of telehealth. First, it suggests that telehealth interest is likely to increase over time as the population as a whole becomes more familiar with and comfortable using different forms of technology. This may be particularly true of social media-based telehealth [44], the newest type of technology included in the survey, and also the technology that respondents reported least confidence and interest in using. Following from this, since our results revealed that technology confidence was modality-specific, whereby confidence using one type of technology was related only to interest in using that same form of telehealth, this suggests that willingness to use telehealth is not restricted to patients who are confident using technology in general. Third, if patients were provided with adequate training and support in using telehealth equipment, they might be more interested in using telehealth. Finally, as some of telehealth’s advantages are realized and other disadvantages are dispelled through telehealth use, the strength of this effect may increase. There is good reason to expect such positive experiences of telehealth, since the majority of telehealth research that asks about patient satisfaction does report fairly high levels of satisfaction [45]. Our study is no exception; high levels of satisfaction with past NHS Direct use—a form of telehealth—were reported. Positive experiences with telehealth may stimulate interest in using additional forms of telehealth, in an upward spiraling effect.

This research suggests that many people with chronic diseases are interested in using telehealth, regardless of their health status and age, and they have the technology available to them. This interest can be increased by helping people gain confidence in using technologies, highlighting the perceived advantages of telehealth, and dispelling or addressing concerns about perceived disadvantages. Based on our findings, future telehealth interventions would be best received by patients if delivered via phone or over email and static forms of Internet, rather than social media. This is because the results show that these forms of technology are readily available, patients are confident using them, and patients are most interested in telehealth delivered via these means.

## Multimedia Appendix 1

Subset of constructed questionnaire items.

## Abbreviations

CVD: cardiovascular disease
GP: general practitioner
MCS: Mental Component Summary
NHS: National Health Service
PCS: Physical Component Summary
SF-12v2: Short-Form Health Survey, version 2
