# Supplementary material for: Are People With Chronic Diseases Interested in Using Telehealth? A Cross-Sectional Postal Survey
Source: J Med Internet Res. 2014 May 8;16(5):e123. doi: 10.2196/jmir.3257 (PMC4034113; doi:10.2196/jmir.3257)
Supplement: Supplementary file 1 [file jmir_v16i5e123_app1.pdf]

Subset of constructed questionnaire items

Socio-demographics items:

1) Are you male or female?

- ☐1 Male
- ☐2 Female

2) What is your ethnic group? (please tick one box only)

- ☐1 White
- ☐2 Mixed
- ☐3 Asian or Asian British
- ☐4 Black or Black British
- ☐5 Any other ethnic group (please describe)

3) How old are you?

- ☐1 18-29yrs
- ☐2 30-44yrs
- ☐3 45-59yrs
- ☐4 60-74yrs
- ☐5 75+yrs

4) Which one of these best describes your current situation? (please tick one box only)

- ☐1 Full-time paid work (30 hours or more each week)
- ☐2 Part-time paid work (under 30 hours each week)
- ☐3 Full-time education at school, college or university
- ☐4 Unemployed
- ☐5 Unable to work due to long term illness/disability
- ☐6 Fully retired from work
- ☐7 Looking after the home
- ☐8 Doing something else (please describe)

5) **Which of these qualifications do you have?** (please tick all the qualifications that apply, or if not specified, tick the nearest equivalent)

- ❑ 1 O levels, CSEs, GCSEs , O grades, or Standard grades
- ❑ 2 A levels, AS levels, Higher School Certificate or Highers (Scotland)
- ❑ 3 Degree or higher degree
- ❑ 4 No qualifications
- ❑ 5 NVQ Levels 1-3/GNVQ
- ❑ 6 NVQ levels 4-5, HNC, HND
- ❑ 7 Other qualifications (for example City and Guilds, RSA/OCR, BTEC/Edexcel)

6) **How do you and your household occupy your accommodation?** (please tick one box)

- ❑ 1 Own it outright
- ❑ 2 Buying it with the help of a mortgage or loan
- ❑ 3 Pay part rent and part mortgage (shared ownership)
- ❑ 4 Rent it
- ❑ 5 Live rent free (including rent free in relative's/friend's property; excluding squatting)
- ❑ 6 Squatting

Access Difficulties:

Service Delivery items:

1) **Sometimes people find it hard to get the health support and advice they would like. Have you had any difficulty with the following?**

	No Difficulty	Some Difficulty	Lots of Difficulty
a. Making appointments for days and times that suit you	❑ 1	❑ 2	❑ 3
b. Making appointments with the particular health professionals that you want to see	❑ 1	❑ 2	❑ 3
c. Having to wait past your appointment time to be seen	❑ 1	❑ 2	❑ 3

d. Getting care and support at the times when you feel you need it most	❑ 1	❑ 2	❑ 3
e. Getting the <u>amount</u> of care and support you feel you need from health professionals (for example, having long enough appointments and being able to see them often enough)	❑ 1	❑ 2	❑ 3
f. Getting the quality of care you want from health professionals	❑ 1	❑ 2	❑ 3
g. Getting enough information about your health and the services available to support you	❑ 1	❑ 2	❑ 3

Physical Access items:

1) Sometimes people find it hard to get the health support and advice they would like. Have you had any difficulty with the following?

	No Difficulty	Some Difficulty	Lots of Difficulty
a. Getting to appointments outside of your home, due to your <u>physical health</u>	❑ 1	❑ 2	❑ 3
b. Getting to appointments outside of your home, due to <u>psychological or emotional difficulties</u>	❑ 1	❑ 2	❑ 3
c. Getting to appointments outside of your home, due to <u>difficulties with transport and travel</u>	❑ 1	❑ 2	❑ 3
d. Cost of transport and travel to get to appointments	❑ 1	❑ 2	❑ 3

Technology-related Factors:

Technology Availability items (Phone (items a-b), Email/Internet (items c-d)):

1) Do you have any of the following <u>easily available</u> for you to use? (For example, at home, at work or at the home of friends or family members) Please tick all boxes that apply

a. A telephone (landline)	❑ 1	c. Internet access	❑ 3
b. A mobile phone	❑ 2	d. A personal e-mail address	❑ 4

Technology Confidence items (Phone Confidence (items a-c), Email/Internet Confidence (items d-f), Social Media Confidence (items g-i)):

2) How confident do you feel about doing the following?

	Nota at all confident	Quite Confident	Extremely confident	I have never tried this	I don't know what this is
a. Using a telephone (landline)	❑ 1	❑ 2	❑ 3	❑ 1	❑ 1
b. Using a mobile phone for phone calls	❑ 1	❑ 2	❑ 3	❑ 1	❑ 1
c. Using a mobile phone to send and receive text messages	❑ 1	❑ 2	❑ 3	❑ 1	❑ 1
d. Using a computer	❑ 1	❑ 2	❑ 3	❑ 1	❑ 1
e. Sending and receiving e-mails	❑ 1	❑ 2	❑ 3	❑ 1	❑ 1
f. Finding out information using the internet	❑ 1	❑ 2	❑ 3	❑ 1	❑ 1
g. Using a 'chat room' on the internet	❑ 1	❑ 2	❑ 3	❑ 1	❑ 1
h. Using social networking sites on the internet, for example 'Facebook'	❑ 1	❑ 2	❑ 3	❑ 1	❑ 1
i. Using 'live messaging' online, for example 'Windows Live Messenger'	❑ 1	❑ 2	❑ 3	❑ 1	❑ 1

Telehealth Advantages & Disadvantages items:

3) How much do you agree or disagree with the following possible advantages:

	Strongly disagree	Disagree	Uncertain	Agree	Strongly agree
a. Getting support in this way would help me to feel more independent	❑ 1	❑ 2	❑ 3	❑ 4	❑ 5
b. I would like being able to choose to get support at times that are best for me	❑ 1	❑ 2	❑ 3	❑ 4	❑ 5
c. I would like being able to get support in my own home	❑ 1	❑ 2	❑ 3	❑ 4	❑ 5
d. It would make me feel special to be getting 'extra' support in this way	❑ 1	❑ 2	❑ 3	❑ 4	❑ 5
e. I would find it reassuring to be able to get support when I feel that I need it most	❑ 1	❑ 2	❑ 3	❑ 4	❑ 5
f. I could save money by not having to travel to appointments	❑ 1	❑ 2	❑ 3	❑ 4	❑ 5

	Strongly disagree	Disagree	Uncertain	Agree	Strongly agree
g. Getting support with my health by phone or computer would be valuable to me	☐ 1	☐ 2	☐ 3	☐ 4	☐ 5

4) How much do you agree or disagree with the following possible disadvantages:

	Strongly disagree	Disagree	Uncertain	Agree	Strongly agree
a. I would worry about relying too much on the technology	☐ 1	☐ 2	☐ 3	☐ 4	☐ 5
b. I would dislike being unable to see the person face-to-face	☐ 1	☐ 2	☐ 3	☐ 4	☐ 5
c. I would not want to discuss sensitive issues over the phone or using a computer	☐ 1	☐ 2	☐ 3	☐ 4	☐ 5
d. I would be concerned about the security of the information that I give	☐ 1	☐ 2	☐ 3	☐ 4	☐ 5
e. Getting support in this way would make me feel anxious about my health	☐ 1	☐ 2	☐ 3	☐ 4	☐ 5
f. I would worry about the possibility of the equipment not working	☐ 1	☐ 2	☐ 3	☐ 4	☐ 5
g. I would dislike speaking to someone other than a doctor about my health	☐ 1	☐ 2	☐ 3	☐ 4	☐ 5

Past Telehealth Satisfaction item:

1) Thinking about the times when you have used NHS Direct services in the past, overall how satisfied have you been with the advice and support they have provided?

Not at all	A little bit	Moderately	Quite a bit	Extremely
☐ 1	☐ 2	☐ 3	☐ 4	☐ 5

Interest in using Telehealth items (Phone-based (items a-c), Email/Internet-based (items d-f), Social Media-based (items g-i)):

1) How strongly would you be <u>interested</u> in using the following to get support with your health from NHS Direct?

	Very interested	Fairly interested	Not at all interested	I don't know what this is
a. Using a telephone (landline)	❑ 3	❑ 2	❑ 1	❑ 1
b. Using a mobile phone for phone calls	❑ 3	❑ 2	❑ 1	❑ 1
c. Using a mobile phone to send and receive text messages	❑ 3	❑ 2	❑ 1	❑ 1
d. Using a computer	❑ 3	❑ 2	❑ 1	❑ 1
e. Sending and receiving e-mails	❑ 3	❑ 2	❑ 1	❑ 1
f. Finding out information using the internet	❑ 3	❑ 2	❑ 1	❑ 1
g. Using a 'chat room' on the internet	❑ 3	❑ 2	❑ 1	❑ 1
h. Using social networking sites on the internet, for example 'Facebook'	❑ 3	❑ 2	❑ 1	❑ 1
i. Using 'live messaging' online, for example 'Windows Live Messenger'	❑ 3	❑ 2	❑ 1	❑ 1
